# High Sensitivity Refractometer Based on Reflective Smf-Small Diameter No Core Fiber Structure

**DOI:** 10.3390/s17061415

**Published:** 2017-06-16

**Authors:** Guorui Zhou, Qiang Wu, Rahul Kumar, Wai Pang Ng, Hao Liu, Longfei Niu, Nageswara Lalam, Xiaodong Yuan, Yuliya Semenova, Gerald Farrell, Jinhui Yuan, Chongxiu Yu, Jie Zeng, Gui Yun Tian, Yong Qing Fu

**Affiliations:** 1 Laser Fusion Research Center, China Academy of Engineering Physics, Mianyang 621900, China; zhougr@caep.cn (G.Z.); lh_liuhao_1989@126.com (H.L.); niulf12@lzu.edu.cn (L.N.); xdyuan@caep.cn (X.Y.); 2 Department of Mathematics, Physics and Electrical Engineering, Northumbria University, Newcastle Upon Tyne NE1 8ST, UK; rahul.kumar@northumbria.ac.uk (R.K.); wai-pang.ng@northumbria.ac.uk (W.P.N.); nageswara.lalam@northumbria.ac.uk (N.L.); richard.fu@northumbria.ac.uk (Y.Q.F.); 3 Photonics Research Centre, Dublin Institute of Technology, Dublin 8, Ireland; yuliya.semenova@dit.ie (Y.S.); gerald.farrell@dit.ie (G.F.); 4 State Key Laboratory of Information Photonics and Optical Communications, Beijing University of Posts and Telecommunications, Beijing 100876, China; yuanjinhui81@bupt.edu.cn (J.Y.); cxyu@bupt.edu.cn (C.Y.); 5 State Key Laboratory of Mechanics and Control of Mechanical Structures, Nanjing University of Aeronautics and Astronautics, Nanjing 210016, China; Jie.Zeng@newcastle.ac.uk; 6 School of Electrical, Electronic and Computer Engineering, Newcastle University, Newcastle Upon Tyne NE1 7RU, UK; g.y.tian@newcastle.ac.uk

**Keywords:** optical fiber sensor, refractometer, single mode-multimode-single mode (SMS) structure, no core fiber

## Abstract

A high sensitivity refractive index sensor based on a single mode-small diameter no core fiber structure is proposed. In this structure, a small diameter no core fiber (SDNCF) used as a sensor probe, was fusion spliced to the end face of a traditional single mode fiber (SMF) and the end face of the SDNCF was coated with a thin film of gold to provide reflective light. The influence of SDNCF diameter and length on the refractive index sensitivity of the sensor has been investigated by both simulations and experiments, where results show that the diameter of SDNCF has significant influence. However, SDNCF length has limited influence on the sensitivity. Experimental results show that a sensitivity of 327 nm/RIU (refractive index unit) has been achieved for refractive indices ranging from 1.33 to 1.38, which agrees well with the simulated results with a sensitivity of 349.5 nm/RIU at refractive indices ranging from 1.33 to 1.38.

## 1. Introduction

Optical fiber sensors have shown great potential for different applications such as detection of biomolecules, measurements of the concentrations of various chemicals, structural health monitoring (SHM) of vital civil engineering structures and composite materials, power systems condition monitoring, and many others due to their inherent advantages such as high sensitivity, low cost, immunity to electromagnetic interference, good corrosion resistance, durability, flexibility, small size, and capability for remote operation [1,2,3,4,5,6,7]. Refractive index (RI) sensing is a basis for many of the fiber based sensing applications, such as medical diagnostics, chemical concentration detection, and biomolecule sensing. To date, many optical fiber refractometer configurations have been studied, including a fiber grating and ring resonance [8,9,10,11,12], single mode-multimode-single mode (SMS) fiber structures [13,14], microfiber interferometers [15,16,17,18], photonic crystal fibers [19,20], surface plasmon resonance fiber sensors [21,22,23], and microstructure tapered fibers [24,25,26,27]. All the above sensing configurations are called evanescent sensors, in which the evanescent field of a waveguide extends into the sensing environment and directly interacts with it, typically resulting in a very high RI sensitivity. However, evanescent sensors are usually associated with complicated fabrication processes or expensive fabrication equipment such as excimer lasers or fiber tapering systems, and hence suffer from relatively high cost [28,29].

Compared to the technologies above, an SMS fiber structure based refractometer has the additional advantages of easy fabrication and low cost. The operating principle of the SMS fiber structure based refractometer is based on multimode interference between modes within a no-core/small-core fiber, which can be influenced easily by the surrounding RI [30,31,32,33,34,35,36,37]. In the previous report [1], in order to fabricate such an SMS based RI sensor, it was necessary to remove the cladding of the multimode fiber (MMF) by means of chemical etching. This additional fabrication step may cause some problems such as difficulty of control over the etching process, roughness of the etched fiber surface, and environmental and health hazards due to the use of etching acids. To avoid the need for chemical etching, a commercially available small core single mode fiber (SCSMF) has been demonstrated to be a good candidate to replace the etched no-core MMF as a sensing probe but with limited sensitivity [33]. In this paper, we propose a small diameter no-core fiber (SDNCF) with a gold layer on the end face as a superior alternative to the SCSMF for RI sensing with improved sensitivity. In addition, the configuration of the sensor is that of an endpoint sensor, whereas traditional SMS based sensors acts as inline sensors. This is an advantage as endpoint sensors are easier to utilize in many applications, for example when functionalized with specific compounds to sense chemical or gaseous measurands.

## 2. Theory and Simulation

**Figure 1:**
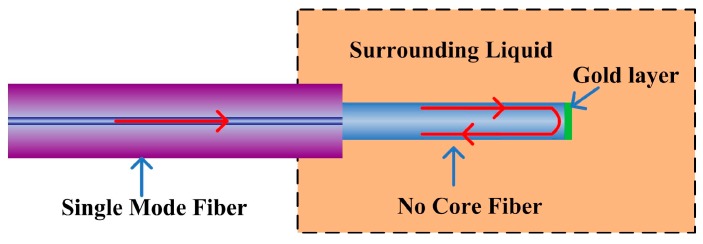
Schematic diagram of the proposed fiber structure as a refractometer.

The schematic configuration of the proposed fiber structure is shown in Figure 1. From the schematic diagram in Figure 1, it can be seen that the surrounding liquid sample under testing effectively acts as a cladding of the SDNCF. As the input light injected from the single mode fiber (SMF) into the SDNCF, multiple modes are excited and propagate within the SDNCF. The multiple modes of the SDNCF are eventually reflected back by the end face of the SDNCF and coupled back to the input SMF which also acts as the output fiber for the sensor.

If the SMF and SDNCF are ideally aligned, the input field at the interface between SMF and SDNCF is circular symmetry where only *LP*_0m_ modes will be excited in the SDNCF when light travels from SMF to SDNCF. Assuming that the SMF section supports the fundamental mode with a field distribution *E*(*r,*0), the *m*th eigenmode field profile within the SDNCF is *ψ_m_*(*r*), the input field can be decomposed into the eigenmodes *LP*_0m_ in the SDNCF [38,39,40].

(1) $$E\left(r,0\right)=\overset{M}{\underset{m=1}{\sum }}b_{m}\psi_{m}\left(r\right)\;$$

The field at the end surface of SDNCF can be written as

(2) $$E\left(r,z\right)=\overset{M}{\underset{m=1}{\sum }}b_{m}\psi_{m}\left(r\right)\exp\left(j\beta_{m}z\right)$$

where *β_m_* is the propagation constant of the *m*th eigenmode of the SDNCF, propagation distance *z* is the length of the SDNCF, and *bm* is the excitation coefficient of the *m*th order mode of the SDNCF which can be written as

(3) $$b_{m}=\frac{\int_{0}^{\infty }E\left(r,0\right)\psi_{m}\left(r\right)rdr}{\int_{0}^{\infty }E\left(r,0\right)E\left(r,0\right)rdr}$$

when the light is reflected back by the end face of the SDNCF, the additional reflection coefficient $\Gamma_{m}$ is introduced to the field *E*(*r,z*) and the field at the output (at the input position of the SDNCF) of the sensor can be expressed as

(4) $$E^{\prime }\left(r,0\right)=\overset{M}{\underset{m=1}{\sum }}\Gamma_{m}E\left(r,z\right)\exp\left(j\beta_{m}z\right)$$

where $\Gamma_{m}$ is the reflectivity of the end face of the SDNCF for each mode. The output power of the structure $P_{out}\left(z\right),$ can thus be expressed as

(5) $$P_{out}\left(z\right)=\frac{{\left|\int_{0}^{\infty }E^{\prime }\left(r,z\right)E\left(r,0\right)rdr\right|}^{2}}{\int_{0}^{\infty }{\left|E\left(r,z\right)\right|}^{2}rdr\int_{0}^{\infty }{\left|E\left(r,0\right)\right|}^{2}rdr}$$

when the RI of the surrounding liquid changes, the effective RI of the cladding of the SDNCF changes as well, which results in the change of *ψ_m_*(*r*) and hence changes the excitation coefficients of each of the modes *b_m_* in Equation (3) and ultimately leads to a change in the optical output of the fiber structure in Equation (5).

**Figure 2:**
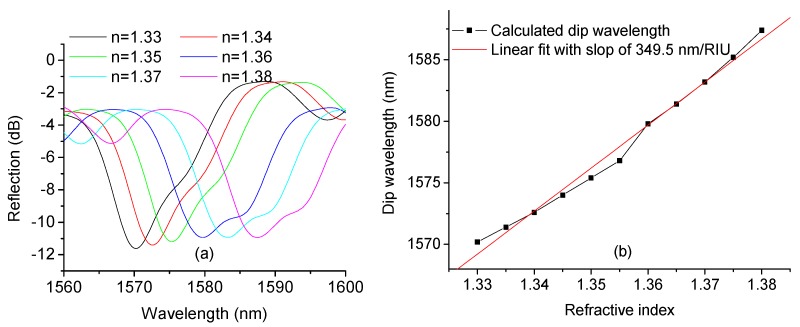
(**a**) Simulated spectral response at different surrounding RIs; and (**b**) dip wavelength shift vs. surrounding RI and its linear fit.

Based on the above analysis, numerical simulations were carried out and the simulated spectral responses for surrounding liquids with various refractive indices are shown in Figure 2a. In our simulation, the RIs of the core and cladding of the SMF were set as 1.4504 and 1.4447 respectively and the core diameter was set to 8.2 µm; the SDNCF had a diameter of 55 µm (which matches that of one of the actual SDNCFs available), RI of 1.4504, and length of 15 mm. Since there is a gold layer on the end face, to simplify the simulation, we assume the reflection coefficient Γ=1. Figure 2a shows that as the surrounding RI increases, the wavelength of the sensor shifts to longer wavelengths monotonically. The dip wavelength vs. surrounding RI is plotted in Figure 2b, which shows a good linear fit (with the coefficient of determination R^2^ = 0.988) with a slope of 349.5 nm/RIU indicating that this sensor has substantially improved sensitivity compared to that of previously reported SCSMF sensor (~135 nm/RIU) in [30].

**Figure 3:**
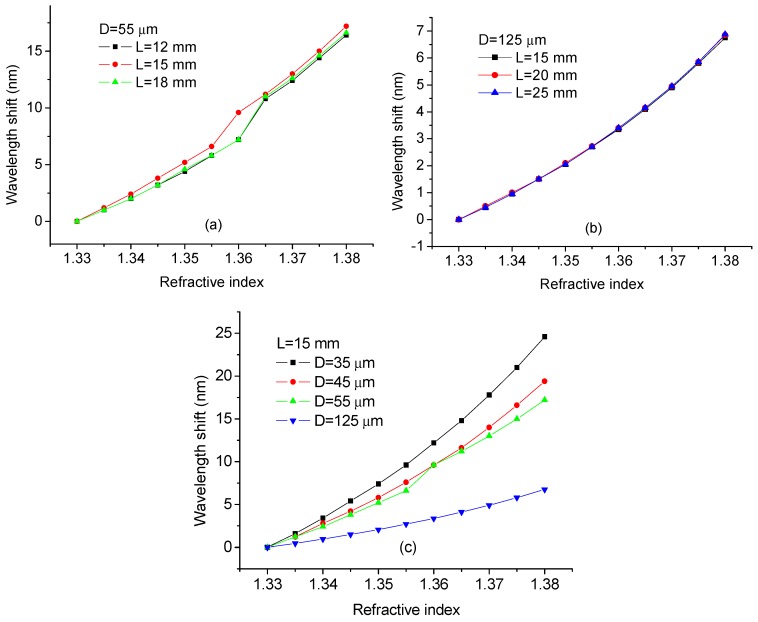
Simulated wavelength shift vs. surrounding RI for different lengths of the SDNCF: (**a**) L = 12, 15, and 18 mm at D = 55 µm; (**b**) L = 15, 20, and 25 mm at D =125 µm; and (**c**) D =35, 45, 55, and 125 µm at L =15 mm.

The influence of the SDNCF length and diameter on the RI sensitivity of the sensor has also been investigated in simulation. The wavelength shifts vs. the length of the SDNCF (D = 55 µm and 125 µm) are shown in Figure 3a,b.

It can be seen for Figure 3a,b that for both diameters of D = 55 µm and 125 µm, the length of the SDNCF has a limited influence on the RI sensitivity, estimated from the linear fit as 336, 349.5, and 340.7 nm/RIU corresponding to the three lengths of 12, 15, and 18 mm with D = 55 µm of SDNCF; and 133.6, 134.7, and 135.7 nm/RIU corresponding to the three lengths of 15, 20, and 25 mm with D = 125 µm of the SDNCF, respectively. The influence of the diameter of the SDNCF on the RI sensitivity is illustrated in Figure 3c. It is easy to see that the smaller the diameter of the SDNCF, the larger is the wavelength shift and hence the higher the sensitivity of the RI sensor. For the SDNCF with a diameter of 35 µm, the estimated sensitivity is as high as 486 nm/RIU, which is a significant improvement compared to that of the 125 µm-diameter SDNCF RI sensor.

## 3. Experimental Investigation

**Figure 4:**
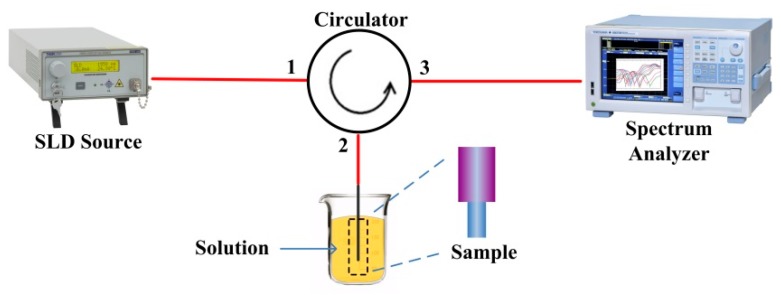
Experimental setup for RI sensing.

For our experiments, the fiber refractometer was fabricated by means of manual fusion splicing of a standard telecommunication fiber SMF28 and a 15 mm long section of SDNCF with a diameter of 55 µm. The fusion arc time and power were adjusted to ensure both a good low loss splice (no obvious camber shape at the splicing interface of the SMF) and mechanical strength of the fusion splice between the SMF and SDNCF. Figure 4 shows a schematic diagram of the experimental setup for RI sensing. Light from an SLD source (Thorlabs S5FC1005S) with a wavelength range of 1450–1650 nm is launched into Port 1 of the circulator, and Port 2 of the circulator is connected to the fiber refractometer. The spectrum analyzer (Yokogawa AQ6370C) connected to Port 3 is used to measure the output spectral response of the fiber refractometer. The fiber structure is fully immersed into an RI liquid sample. All measurements were carried out at room temperature.

**Figure 5:**
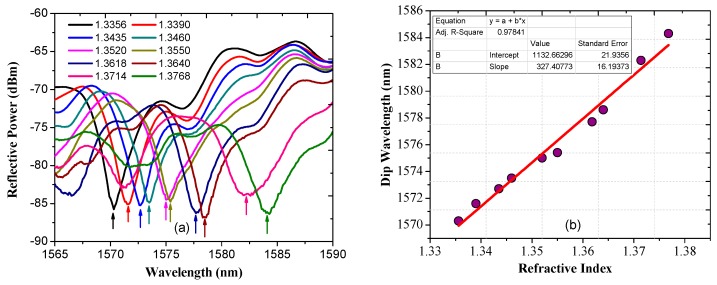
Experimentally measured (**a**) spectral responses; and (**b**) wavelength shifts for the RI sensor with D = 55 µm in various liquids.

Figure 5a shows the measured spectral responses in various RI liquids. These RI liquids were made with different concentration sugar solutions which were calibrated using an Abbe refractometer (Kruess AR4D). The RI values for the RI liquids in our experiment were in the range of 1.33–1.38. As shown in Figure 5a, the spectral response shifts monotonically towards longer wavelengths as the RI increases. Figure 5b shows the dependence of the wavelength of the dip in the spectral response vs. different surrounding RIs. It indicates that the wavelength shift of the sensor’s spectral response exhibits good linearity with the increase of RI. The sensitivity of the fiber sensor is estimated from the graph as 327 nm/RIU, which is very close to the simulated value of 349.5 nm/RIU, indicating that our developed simulation model is reliable.

**Figure 6:**
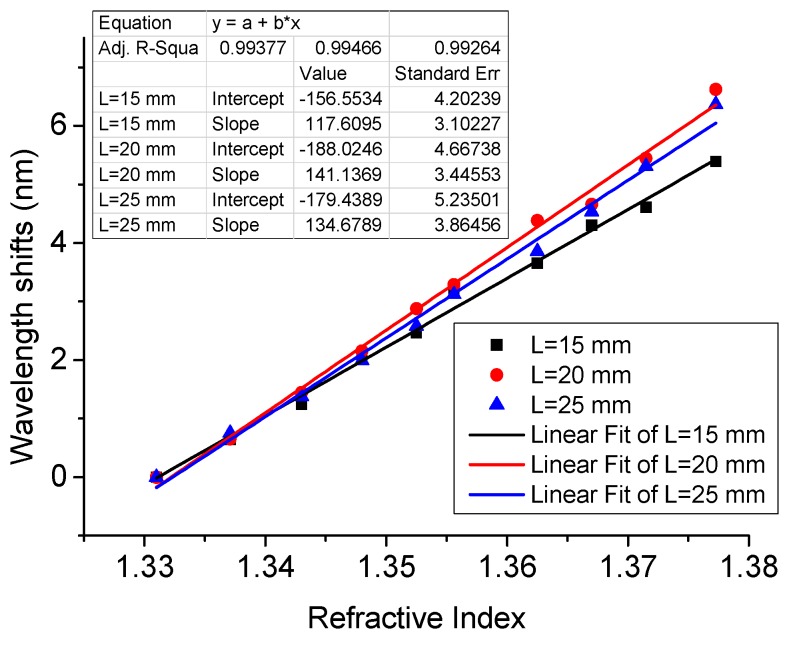
Measured wavelength shifts for RI sensors with L = 15, 20, and 25 mm and D = 125 µm in various liquids.

To investigate the influence of the SDNCF length experimentally, three sensors with different lengths of 15, 20, and 25 mm with D = 125 µm were fabricated and studied. Figure 6 shows the measured wavelength shifts vs. different RI for the three sensors.

Figure 6 shows that the three sensors with lengths of 15, 20, and 25 mm have a sensitivity of 117.6, 141.1, 134.7 nm/RIU respectively, confirming that the length of the SDNCF has a very limited influence on the sensitivity of the RI sensor. The experimental results are also in good agreement with the simulated value of ~134 nm/RIU. The results also show that RI sensor with the larger SDNCF diameter (125 µm) has a smaller sensitivity (maximum 141 nm/RIU) compared to that (~327 nm/RIU) for the smaller SDNCF diameter (55 µm). Table 1 compares the RI sensitivity of refractometric fiber sensors reported previously with our proposed sensor. As one can see from the comparison, RI sensitivity of the proposed structure with SDNCF is the highest as demonstrated experimentally.

## 4. Conclusions

In conclusion, we proposed a new reflective SDNCF based SMS fiber structure for RI sensing. Both simulation and experimental results show that wavelength shift of the refractometer exhibits a good linear relationship with an increase in the RI of the liquid under test. Our simulation results show that the length of SDNCF has a limited influence on the RI sensitivity of the refractometer, but the diameter of SDNCF does have a significant influence on the RI sensitivity. The calculated maximum sensitivity for the RI sensor is 486 nm/RIU for the SDNCF with a diameter of 35 µm in the RI range from 1.33 to 1.38. The measured experimental sensitivity of the fiber refractometer with a SDNCF diameter of 55 µm is 327 nm/RIU, which is 2.4 times higher than that reported in [33], and it also agrees well with the simulation result of 349.5 nm/RIU, indicating that the model developed in this paper is reliable. Compared to other types of fiber RI sensors, the proposed refractometer has the advantages of being an endpoint sensor, which demonstrates a high sensitivity combined with a simple structure and easy fabrication. If an even smaller diameter commercial no-core fiber was used to replace the current 55 µm fiber, the sensitivity of the refractometer could be further improved.

It should be noted that the proposed fiber structure used with an appropriate coating could have a wide range of applications. For example, if a Pt-decorated graphene oxide film is coated on the surface of the no core fiber [41], a highly sensitive ammonia (NH_3_) optical fiber sensor based on the SDNCF can be developed. Another example is the use of a magnetic fluid whose refractive index changes under the influence of magnetic field. Using such a magnetic fluid as a coating, a magnetic field sensor based on the SDNCF could be developed. In summary, the proposed reflective SDNCF based fiber refractometer potentially has a wide range of applications in biology, chemistry and environmental engineering, recognition of bacteria, nuclear leakage monitoring, magnetic field detection, humidity monitoring, and chemical analysis. By properly designing and cascading several SDNCF sections within the sensor structure, it is possible to realize multiple parameters’ detection if different functional layers were deposited on the surface of each of the SDNCF sections.

## Figures and Tables

**Table 1:** Sensitivity for fiber Refractometric sensors.

No.	Type of Sensor	Sensitivity (nm/RIU)	Ref.
1	Tapered single-mode optical fiber	26.087	[25]
2	The thin-core fiber modal interferometers	135.5	[33]
3	The no-core fibers	227.14	[34]
4	Etched multimode fiber	286.2	[31]
5	The small diameter no-core fiber (SDNCF)	327	This work
